# Predictors of new graduate nurses’ health over the first 4 years of practice

**DOI:** 10.1002/nop2.231

**Published:** 2018-12-19

**Authors:** Heather K. Spence Laschinger, Carol Wong, Emily Read, Greta Cummings, Michael Leiter, Maura Macphee, Sandra Regan, Ann Rhéaume‐Brüning, Judith Ritchie, Vanessa Burkoski, Doris Grinspun, Mary Ellen Gurnham, Sherri Huckstep, Lianne Jeffs, Sandra Macdonald‐Rencz, Maurio Ruffolo, Judith Shamian, Angela Wolff, Carol Young‐Ritchie, Kevin Wood

**Affiliations:** ^1^ Arthur and Sonia Labatt Family School of Nursing University of Western Ontario London Ontario Canada; ^2^ University of New Brunswick Fredericton New Brunswick Canada; ^3^ Faculty of Nursing University of Alberta Edmonton Alberta Canada; ^4^ Department of Psychology Faculty of Science Acadia University Wolfville Nova Scotia Canada; ^5^ Centre for Organizational Research and Development Acadia University Wolfville Nova Scotia Canada; ^6^ School of Nursing University of British Columbia Vancouver British Columbia Canada; ^7^ School of Nursing Science Faculty of Health Sciences and Community Services Universite de Moncton Moncton New Brunswick Canada; ^8^ McGill University Health Centre Research Department Montreal Quebec Canada; ^9^ London Health Sciences Centre London Ontario Canada; ^10^ Registered Nurses’ Association of Ontario Toronto Ontario Canada; ^11^ Capital Health District Health Authority Halifax Nova Scotia Canada; ^12^ Victorian Order of Nurses Ottawa Ontario Canada; ^13^ Nursing/Clinical Research Nursing Administration St. Michael's Hospital Toronto Ontario Canada; ^14^ Office of Nursing Policy Health Policy Branch Health Canada Ottawa Ontario Canada; ^15^ Providence Care Kingston Ontario Canada; ^16^ International Council of Nurses Geneva Switzerland; ^17^ Department of Clinical Education, Professional Practice and Integration Fraser Health Surrey British Columbia Canada

**Keywords:** burnout, health, incivility, mental health, new graduate nurses, nursing, occupational coping self‐efficacy, post‐traumatic stress disorder risk, psychological capital

## Abstract

**Aim:**

To examine predictors of Canadian new graduate nurses’ health outcomes over 1 year.

**Design:**

A time‐lagged mail survey was conducted.

**Method:**

New graduate nurses across Canada (*N* = 406) responded to a mail survey at two time points: November 2012–March 2013 (Time 1) and May–July 2014 (Time 2). Multiple linear regression (mental and overall health) and logistic regression (post‐traumatic stress disorder risk) analyses were conducted to assess the impact of Time 1 predictors on Time 2 health outcomes.

**Results:**

Both situational and personal factors were significantly related to mental and overall health and post‐traumatic stress disorder risk. Regression analysis identified that cynicism was a significant predictor of all three health outcomes, while occupational coping self‐efficacy explained unique variance in mental health and work–life interference explained unique variance in post‐traumatic stress disorder risk.

**Why is this research needed?**

- Past research shows that new graduate nurses experience high levels of stress and burnout during their transition to professional practice, factors which are linked with poor employee mental and physical health outcomes.
- As the nursing workforce ages, understanding personal and work environment factors that contribute to new graduate nurses’ health is important to ensure retention of newcomers to the profession.

**What are the key findings?**

- This is one of the first studies to demonstrate that positive work environment factors are associated with better health outcomes for new graduate nurses.
- Results add to growing evidence that development of new graduate nurses’ intrapersonal resources contributes to their health and well‐being during their stressful transition to practice.
- Findings linking incivility to new graduate nurses’ post‐traumatic stress disorder risk add to past research by demonstrating that incivility, while thought to be less severe than bullying, is also psychologically harmful to new graduate nurses.
- While the link between burnout and new graduate nurses’ mental and physical health has been reported in previous studies, ours is the first to show that burnout is positively related to increased risk of post‐traumatic stress disorder in this population.
- Psychological capital and occupational coping self‐efficacy were significantly related to all three health outcomes, highlighting the important role that new graduate nurses’ intrapersonal resources play in protecting their health, thought to be related to their ability to handle work‐related stressors.

**How should the findings be used to influence policy/practice/research/education?**

- The links between authentic leadership and positive, empowering working conditions and new graduate nurses’ health outcomes suggest that situational support for leadership development and positive nursing work environments is important.
- Nurse managers should work to establish and maintain work environments that support the development of new graduate nurses’ intrapersonal resources and foster civility and collegiality.
- Given the powerful negative effect of cynicism on new graduate nurses’ mental and overall health and risk of post‐traumatic stress disorder, education, assessment and prevention of burnout development are critical strategies for nurse managers to maintain a healthy new graduate nurse workforce.

## 1 INTRODUCTION

New graduate nurses often find the first few years of practice challenging and stressful as they transition from school to the workplace (Duchscher, 2008; Rheaume, Clement, & LeBel, 2011). New graduate nurses continue to report high levels of stress, anxiety and burnout during their initial years in the workforce (Casey, Fink, Krugman, & Propst, 2004; Laschinger & Grau, 2012; Parker, Giles, Lantry, & McMillan, 2014), leading many of them to consider leaving the profession in their first 2–5 years of practice (Parker et al., 2014; Rudman, Gustavsson, & Hultell, 2014).

Research findings suggest that unhealthy or stressful nursing work environments have major negative implications for nurses’ health and well‐being over time (Letvak & Buck, 2008; Tucker, Harris, Pipe, & Stevens, 2010). For example, in a recent study by Melnyk, Hrabe, and Szalacha (2013), higher workplace stress was associated with a negative impact on nurses’ mental health, including greater reports of depressive symptoms and anxiety levels. In contrast, supportive work environments positively influence new graduate nurses’ transition to practice (Laschinger, Finegan, & Wilk, 2009; Rheaume et al., 2011) and have been associated with better mental and physical health (Read & Laschinger, 2015). Personal factors also play an important role in determining nurses’ health. For example, stress appraisal and coping strategies influence nurses’ perceptions of stress, which in turn affects their mental and physical health (Chang et al., 2007) and burnout levels (Khamisa, Peltzer, & Oldenburg, 2013). However, few studies have examined how work environment and personal factors influence new graduate nurses’ health outcomes during their transition to practice. Therefore, the aim of the current study was to address the following research question: What are the significant work environment and personal variables that influence new graduate nurses’ mental health, overall health and post‐traumatic stress disorder (PTSD) risk during the first few years of practice?

## 2 BACKGROUND

**Figure 1:**
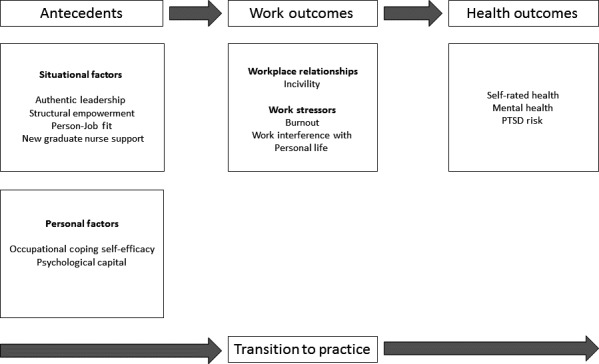
New graduate successful transition model

The guiding framework for this study was The New Graduate Successful Transition Retention Model (Scott, Engelke, & Swanson, 2008; for detailed explanation). This model was chosen because it is evidence‐informed and gives a logical framework to understand the transition experiences of new graduate nurses. As shown in Figure 1, this model identifies personal and situational factors and mediating work environment characteristics which influence new graduate nurses’ mental and overall health and PTSD risk. Personal factors describe the characteristics of individuals, whereas situational factors relate to workplace dynamics. The combination of these factors influences the way new graduate nurses perceive and behave in everyday work experiences (such as bullying/incivility, burnout and work–life balance), which in turn impact health outcomes such as mental health, overall health and PTSD risk.

### 2.1 Antecedents—Situational factors

#### 2.1.1 Authentic leadership

Authentic leadership focuses on honesty, integrity and high moral values to create supportive and positive work environments. Avolio and Gardner (2005) described four core authentic leadership behaviours which are as follows: balanced processing, relational transparency, self‐awareness and an internalized moral perspective. Balanced processing describes a leader's ability to objectively consider all feedback and opinions prior to resolving an important decision (Leroy, Palanski, & Simons, 2012). Authentic leaders show relational transparency when they honestly present themselves to others and model openness and acceptance, promoting an environment where others feel safe in disclosing their ideas, challenges, opinion and goals (Avolio & Gardner, 2005). In authentic leadership theory, high levels of self‐awareness are essential as they provide leaders with insight and understanding of their own strengths and limitations (Kernis, 2003), and their values and beliefs (Avolio & Gardner, 2005). Finally, an internalized moral perspective reflects a consistency between defining a high standard of ethical and moral principles and their subsequent behaviours which reflect these values (Walumbwa, Avolio, Gardner, Wernsing, & Peterson, 2008). Research has demonstrated positive links between nurses’ perceptions of their managers’ authentic leadership behaviours and positive nursing work conditions including work engagement, job satisfaction, trust in management and perceptions of unit care quality (Giallonardo, Wong, & Iwasiw, 2010; Wong, Laschinger, & Cummings, 2010). These studies highlight the key role of authentic leadership in creating desirable workplaces for new graduate nurses.

#### 2.1.2 Structural empowerment

Structural empowerment theory (Kanter, 1993) proposes that when employees have access to four key workplace structures: access to information, access to resources, access to support and access to opportunities to learn and grow, they are empowered to accomplish their work in meaningful ways. Access to information involves making sure that workplace policies, procedures and knowledge about organizational goals are easily accessible to employees. Having access to resources pertains to equipment, supplies and time that an employee needs to accomplish their jobs. Access to support includes the provision of constructive feedback and assistance from senior colleagues and their leader, allowing employees to learn and consolidate their skills and abilities. Lastly, access to opportunities means facilitating employee growth and development through ongoing training, involvement in engaging and challenging work and attendance at professional conferences. Numerous studies have associated structural empowerment with nurses’ work and health outcomes, patient care quality and job satisfaction (Laschinger & Fida, 2015; Wing, Regan, & Spence Laschinger, 2015). Authentic leadership has been linked with structurally empowering working conditions, which may protect against burnout (Laschinger & Grau, 2012). Both structural empowerment and authentic leadership have been related to perceptions of a sense of community at work, which leads to better mental health outcomes (Read & Laschinger, 2015).

#### 2.1.3 Person‐job fit with six areas of work–life

Maslach and Leiter (1997) identified six essential areas of work–life that can enhance employee engagement at work when there is a good match between employees’ expectations and their actual working conditions (also known as person‐job fit). These six areas include workload (job demands), control (autonomy and decision‐making capacity), rewards (financial, social, or personal recognition or compensation), community (quality relationships and sense of comradery at work), fairness (perceived justice) and values congruence (match between employee and organization priorities and values; Maslach & Leiter, 1997). These areas of work–life have been significantly related to structural empowerment (Laschinger, Wong, & Greco, 2006) and authentic leadership (Bamford, Wong, & Laschinger, 2013), and lower levels of job burnout (Brom, Buruck, Horváth, Richter, & Leiter, 2015) and better employee mental health (Laschinger, Borgogni, Consiglio, & Read, 2015).

#### 2.1.4 New graduate nurse support

Several support strategies have been implemented in an attempt to ease new graduate nurses’ transition to practice, an experience which has been reportedly stressful for those trying to navigate this new role (Casey et al., 2004; Salt, Cummings, & Profetto‐McGrath, 2008). These include formal programmes such as nurse residency programs, preceptorships and mentorships and peer support opportunities (Rush, Adamack, Gordon, Lilly, & Janke, 2013). These programmes have had an overall positive impact for new graduate nurses and organizations, such as improved retention rates (with one‐year retention rates for nurses with these formal supports being as high as 90%).

### 2.2 Antecedents—Personal factors

#### 2.2.1 Occupational coping self‐efficacy

Occupational coping self‐efficacy is a person's self‐assessed capability to cope with environmental demands (Pisanti, Lombard, Lucidi, Lazzari, & Bertini, 2008). Individuals’ beliefs of their ability to manage work‐related stress enable them to view these difficulties as positive learning opportunities and remain active and persistent in investing their effort to overcome these challenges (Bandura, 1997). Occupational coping self‐efficacy has been linked to positive and proactive coping (Schwarzer & Knoll, 2003). A previous study showed that new nurses’ occupational coping self‐efficacy had a significant negative effect on burnout and mental health (Laschinger et al., 2015).

#### 2.2.2 Psychological capital

The influence of psychological capital (PsyCap) as personal dispositional factors on nurses’ work experiences has been reported in several studies. Psychological capital (PsyCap) is defined as “an individual's positive psychological state of development that is characterized by: (a) having confidence (self‐efficacy) to take on and put in the necessary effort to succeed at challenging tasks; (b) making a positive attribution (optimism) about succeeding now and in the future; (c) persevering toward goals and, when necessary, redirecting paths to goals (hope) to succeed; and (d) when beset by problems and adversity, sustaining and bouncing back and even beyond (resiliency) to attain success” (Luthans, Avolio, Avey, & Norman, 2007, p. 3). Self‐efficacy refers to an individual's sense of confidence in his/her ability to successfully perform a specific task (Bandura, 1997). Hope describes individuals’ motive to attain their goals (Luthans et al., 2007). Optimism reflects individual's perception that less desirable situations are caused by sources that are external, momentary and situational, compared with more desirable situations that are caused by internal and lasting sources (Luthans & Youssef, 2004). Resilience is defined as a person's ability to recover from hard time (Luthans, Luthans, & Luthans, 2004). Laschinger and Grau (2012) found that PsyCap contributed to enhancing new graduate nurses’ physical and mental well‐being.

### 2.3 Work outcomes–Workplace relationships

#### 2.3.1 Workplace incivility

Workplace relationships play a valuable role in new graduate nurses’ early job and career satisfaction (Winter‐Collins & McDaniel, 2000). Unfortunately, there is growing evidence that workplace mistreatment is prevalent in nursing. For example, Lewis and Malecha (2011) found that 85% of staff nurses in Texas hospitals experienced workplace incivility, while Johnson and Rea (2009) found that 27.5% of new graduate nurses experienced bullying in the first 6 months of their career. Workplace bullying and incivility are costs to employees and organizations and have significant negative effects on nurses’ mental and physical health, evidenced by numerous studies. Incivility, which describes rude, disrespectful and uncaring actions with an ambiguous intent to harm others (Andersson & Pearson, 1999), has been linked to decreased mental health (Laschinger, Wong, Regan, Young‐Ritchie, & Bushell, 2013; Wing et al., 2015) and physical health (Read & Laschinger, 2013) among new graduate nurses. Bullying, which refers to more severe, ongoing and targeted actions intended to harm another individual (Kivimäki, Elovainio, & Vahtera, 2000), has been linked directly to PTSD risk among new graduate nurses (Laschinger & Nosko, 2015), and to poor physical and mental health through its effect on burnout (Laschinger & Grau, 2012; Laschinger, Grau, Finegan, & Wilk, 2010). Together, these studies highlight the damaging effects of workplace bullying and incivility on new graduate nurses’ health during their transition to the workforce. These forms of workplace mistreatment can be emotionally and physically draining, causing high levels of stress, fear and anxiety, leading to poor mental and overall health and PTSD risk. Therefore, we expected that experienced bullying and incivility would have significant negative effects on new graduate nurses’ health outcomes.

### 2.4 Work Outcomes–Work Stressors

#### 2.4.1 Burnout

Burnout refers to a psychological response to chronic exposure to stressful working conditions characterized by emotional exhaustion (feeling too tired to invest the energy needed to fully respond and engage in one's work) and feeling cynical (questioning the meaning and importance of one's work; Leiter & Maslach, 2004). Both components of job burnout have been negatively associated with new graduate nurses’ health (Laschinger & Fida, 2014a; Laschinger & Grau, 2012). In the present study, we examined the role of each of these aspects of job burnout on nurses’ health outcomes, taking into consideration other situational and personal factors.

### 2.5 Nurses’ health outcomes

A healthy nursing workforce is an essential component of sustainable healthcare delivery; thus, new graduate nurses’ physical and mental health, including PTSD risk, are important outcomes. Recent studies have shown that work environment factors (e.g., positive leadership, empowering work environments) and personal factors (e.g., PsyCap, occupational coping self‐efficacy) have positive effects on new graduate nurses’ mental health (Laschinger & Grau, 2012; Laschinger et al., 2013). On the contrary, burnout, incivility and bullying have been linked to poor mental and overall health among this population (Laschinger et al., 2010; Wing et al., 2013).

#### 2.5.1 Work interference with personal life

Work–life balance is an important issue for many employees (Hays Specialist Recruitment Limited, 2010; Jamieson, Kirk, & Andrew, ) and is highly valued by Generation Y workers (McCrindle, 2006; McCrindle & Pleffer, 2008), who predominantly make up the current new graduate nurse workforce. Compared with older workers, younger employees tend not to view work as their life and desire work that is compatible with their lifestyle and other commitments. Jamieson et al. ( ) found that Generation Y nurses have a strong preference for shift schedules that complement their personal lives along with workloads that do not leave them too exhausted to enjoy their time off. In another study, Generation Y nurses identified better working hours and work–life balance as factors that they would like to change about their profession (Jamieson et al., ). Work–life interference, which occurs when employees feel that their job is intruding on their personal life, is an indicator of poor work–life balance and has been linked to increased burnout among new graduate nurses, resulting in increased job turnover intentions (Boamah & Laschinger, 2015). Other studies have shown strong associations between work–life conflict and nurses’ mental and physical health (Munir, Nielsen, Garde, Albertsen, & Carneiro, 2012). Thus, work–life interference is an important factor influencing new graduate nurses’ health.

### 2.6 Summary

As many countries around the world currently face an ageing nursing workforce and a shortage of qualified nurses, retaining newly graduated nurses is a pressing concern for healthcare organizations. Therefore, understanding factors affecting the mental and physical health of this cohort of nurses is important. The New Graduate Successful Transition Model (Scott et al., 2008) was used as a theoretical framework to describe the health outcomes which influence new graduate transition and retention during the first 3 years of practice. In this study, we examined self‐rated overall health, mental health and PTSD.

## 3 THE STUDY

### 3.1 Design

This aim of this study was to describe the work–life experiences and health of new graduate nurses across Canada over 1 year and examine how situational and personal factors influence their mental and overall health and PTSD risk caused by exposure to incivility at work. Therefore, a time‐lagged mail survey was conducted.

### 3.2 Method

#### 3.2.1 Participants

Each of the 10 provinces in Canada was asked to provide a sample of up to 400 registered nurses newly registered in the last 3 years. In total 3,743 nurses were eligible and received a survey at Time 1 (November 2012–March 2013). Of these, 1,020 nurses returned a completed Time 1 questionnaire (response rate = 27.3%). At Time 2 (May–July 2014), 406 of the 1,020 nurses who completed a survey at Time 1 returned a completed questionnaire (response rate = 39.8%), resulting in a final data set of matched Time 1 and Time 2 data for 406 new graduate nurses.

#### 3.2.2 Data collection

Data collection procedures recommended by Dillman, Smyth, and Christian (2014) were used to enhance response rates. At both Time 1 and Time 2, eligible participants received a survey package in the mail to their home address that included a study letter of information, a survey booklet with a unique PIN, a prepaid return envelope and a $2 coffee card. Four weeks after the initial mailing a reminder letter was sent to participants who had not yet responded. Finally, a second survey package was sent to remaining non‐responders 4 weeks after that.

#### 3.2.3 Instruments

Standardized questionnaires with acceptable psychometric properties and demonstrated construct validity were used to measure major study variables (Table 1). Each of these instruments has been well‐validated and used in past studies.

**Table 1:** Study instruments

Variable	Instrument	Items	Range	*α*	Validity
Antecedents					
Situational variables					
Authentic leadership	Authentic leadership questionnaire (Walumbwa et al., 2008)	16	0–4	0.96	Validity of the ALQ has been supported by a number of studies (see Roof, 2014; for a review)
Transparency		4	0–4	0.87	
Ethical/Moral		4	0–4	0.87	
Balanced processing		4	0–4	0.84	
Self‐awareness		4	0–4	0.92	
Structural empowerment	Conditions for work effectiveness questionnaire‐II (Laschinger et al. 2001)	12	4–20	0.85	Confirmatory factor analyses for the CWEQ‐II has supported the validity of this instrument (Laschinger, Finegan, Shamian & Wilk, 2001)
Opportunity		3	1–5	0.85	
Support		3	1–5	0.83	
Information		3	1–5	0.84	
Resources		3	1–5	0.83	
Person‐job fit	Areas of work–life scale (AWS; Leiter & Maslach, 2004)	20	1–5	0.81	The AWS has demonstrated a consistent factor structure across samples, supporting its construct validity (Leiter & Maslach, 2004, Leiter, Gascon, & Marinez‐Jarreta, 2010)
Workload		3	1–5	0.69	
Control		5	1–5	0.52	
Reward		3	1–5	0.77	
Community		3	1–5	0.72	
Fairness		3	1–5	0.41	
Values		3	1–5	0.76	
New graduate nurse support	The casey‐fink graduate nurse experience survey: supportive environment (Casey et al., 2004)	9	1–4	0.86	Content and criterion validity of this instrument was supported in the initial study (Casey et al., 2004).
Personal variables					
Occupational coping self‐efficacy	Occupational coping self‐efficacy for nurses (OCSE‐N; Pisanti et al., 2008)	9	1–5	0.84	EFA and CFA demonstrated support for the construct validity of this instrument. Criterion validity was also supported (Pisanti et al., 2008)
Psychological capital	Psychological capital questionnaire (Luthans et al., 2007)	12	1–6	0.88	The construct validity of this instrument has been supported by CFA. Convergent validity and discriminant validity have also been supported (Luthans et al., 2007)
Hope		4	1–6	0.82	
Resiliency		3	1–6	0.74	
Optimism		2	1–6	0.72	
Resiliency		3	1–6	0.81	
Work outcomes					
Workplace relationships					
Incivility	Straightforward workplace incivility scale (Leiter & Day, 2013)				CFA showed that a 3‐factor structure yields a better fit than a one‐factor structure, supporting the construct validity of the scale (Portoghese, Galletta, Leiter, & Campagna, 2015)
Supervisor Incivility		5	0–6	0.90	
Co‐Worker Incivility		5	0–6	0.91	
Physician Incivility		5	0–6	0.91	
Work stressors					
Burnout	The maslach burnout inventory (Maslach, Jackson, & Leiter, 1996)				CFA supported the 3‐factor structure of the MBI (Maslach et al., 1996). The MBI is a well‐established questionnaire that has been used in numerous studies and has consistently demonstrated convergent, discriminant and criterion validity. Note that we only used two of the three subscales in this study
Emotional exhaustion		5	0–6	0.93	
Cynicism		5	0–6	0.91	
Work interference with personal life	Work interference with personal life (Hayman, 2005)	7	1–7	0.92	CFA has supported the construct validity of this instrument (Hayman, 2005)
Health outcomes					
Self‐rated Health	The SF‐36 General Survey (Ware, Kosinski, Dewey, & Gandek, 2000)	1	1–4	–	The SF‐36 is a well‐established health questionnaire that has demonstrated strong construct validity using CFA in countries worldwide (Keller et al., 1998)
Mental health	General health questionnaire‐12 (Goldberg & Williams, 1988)	12	1–4	0.83	The construct validity of the GHQ‐12 has been demonstrated (Goldberg & Williams, 1988). This questionnaire has also demonstrated criterion validity (Lundin, Hallgren, Theobald, Hellgren, & Torgén, 2016)
PTSD risk	Primary Care Post‐Traumatic Stress Disorder Screen (PC‐PTSD; Prins et al., 2003)	6	0–1	–	The PC‐PTSD has demonstrated high construct and criterion validity and has been shown to better predict PTSD than other screening tools for PTSD (Prins et al., 2003)

Note

All scales are scored such that higher scores represent higher levels of the variable being measured with the exception of mental health symptoms where higher scores represent lower levels of mental health symptoms.

### 3.3 Analysis

SPSS version 22.0 (IBM, 2014) was used to conduct descriptive and inferential statistics. Paired‐samples *t* tests and chi‐square analyses were used to compare differences between Time 1‐Time 2 major study variables. Relationships between main study variables were assessed using Pearson's correlations and biserial correlations (PTSD). Regression analysis was used to examine the unique variance that each of the predictor variables accounted for in the three health outcomes. Two separate multiple linear regression analyses were performed to assess the impact of Time 1 predictor variables on Time 2 mental health and overall health (continuous variables). Logistic regression analysis was performed to examine the effects on the presence of PTSD risk at Time 2 (dichotomous variable). For each of the multiple linear regression models, *r*‐square was calculated. Nagelkerke's *r*‐square, which is the coefficient of determination for a logistical regression analysis, was calculated to provide a measure of the explanatory power of the logistic regression model (Nagelkerke, 1991).

### 3.4 Ethics

Institutional Research Ethics Committee was obtained by the principal investigator before commencing the study. Only registered nurses who had previously given consent to their provincial regulatory body were included in the sample obtained for the study. The mail‐out package sent to potential participants included a detailed letter of information outlining the purpose of the study, possible risks and benefits of participating and made it clear that participation was optional. Returning a completed survey indicated informed consent.

## 4 RESULTS

At Time 1, participants were mostly female (91.8%) with a mean age of 27.68 (6.88). Most were baccalaureate‐prepared nurses (94.1%) working full‐time (57.1%) or part‐time (32.5%). Medical or surgical units were the most common specialty area of practice (51.7%), while 16.3% worked in critical care, 11.6% in maternal or child areas and 5.4% in mental health. An additional 14.5% indicated their specialty as “other.” Participant characteristics for Time 1 and Time 2 are presented in Table 2.

**Table 2:** Participant characteristics (*N* = 406)

	Time 1 *Mean* (*SD*)	Time 2 *Mean* (*SD*)
Age	27.68 (6.88)	29.2 (6.99)
Years of experience as RN	1.17 (0.52)	2.65 (0.53)

	*N* (%)	*N* (%)
Gender		
Female	369 (91.8)	368 (91.3)
Male	33 (8.2)	35 (8.6)
Highest degree received		
BScN	382 (94.1)	379 (93.3)
MScN	0 (0)	3 (0.7)
College Diploma	23 (5.7)	22 (5.4)
Employment status		
Full‐time	232 (57.1)	253 (62.3)
Part‐time	132 (32.5)	122 (30.0)
Casual	40 (9.9)	29 (7.1)
Hours per week		
Less than 20 hr	11 (2.7)	9 (2.2)
20–39 hr	210 (51.7)	242 (59.6)
More than 39 hr	178 (43.8)	146 (36.0)
Unit specialty		
Medical‐surgical	210 (51.7)	176 (43.3)
Critical care	66 (16.3)	88 (21.7)
Maternal–Child	47 (11.6)	49 (21.1)
Mental health	22 (5.4)	28 (6.9)
Other hospital unit	61 (14.5)	65 (15.8)

### 4.1 Descriptive results

Means and standard deviations for the major study variables, in addition to paired‐samples *t* tests examining the change over time, are provided in Table 3.

**Table 3:** Descriptive statistics for study variables at Time 1 and Time 2

Variable	Score range	Time 1 *Mean* (*SD*)	Time 2 *Mean* (*SD*)	*t*‐value	*p*
Occupational coping self‐efficacy	1–5	3.59 (0.56)	3.62 (0.54)	−1.268	0.206
Psychological capital	1–6	4.56 (0.64)	4.65 (0.66)	−3.079	0.002^a^
Authentic leadership	0–4	2.64 (0.87)	2.51 (0.90)	2.675	0.008^a^
Structural empowerment	4–20	13.73 (2.46)	13.39 (2.44)	2.721	0.007^a^
Person‐job fit	1–5	3.28 (0.45)	3.22 (0.48)	2.497	0.013^a^
New graduate support	1–4	3.23 (0.49)	3.23 (0.47)	0.331	0.741
Work–life interference	1–7	3.67 (1.40)	3.77 (1.43)	−1.533	0.126
Supervisor incivility	0–6	0.71 (1.03)	0.72 (0.98)	−0.089	0.929
Co‐worker incivility	0–6	0.91 (1.01)	0.91 (1.06)	−0.038	0.970
Physician incivility	0–6	1.25 (1.24)	1.22 (1.24)	0.193	0.847
Emotional exhaustion	0–6	3.28 (1.50)	3.30 (1.49)	−0.374	0.709
Cynicism	0–6	1.55 (1.48)	1.79 (1.56)	−3.360	0.001^a^
Mental health symptoms	1–4	2.76 (0.44)	2.81 (0.48)	−1.901	0.058
Overall health	1–4	3.12 (0.65)	3.12 (0.62)	−0.151	0.880

Variable	Score Range	*N*	%	*N*	%	*χ* ^2^	*p*
PTSD (Reported 3 or 4 symptoms)	0–1	89	21.9	96	23.6	87.991	<0.001^a^
Nightmares/unwanted thoughts	0–1	211	52.0	213	52.5	33.974	<0.001^a^
Avoid thoughts/situations	0–1	195	48.0	196	48.3	45.312	<0.001^a^
Constantly on guard, watchful	0–1	86	21.2	71	17.7	41.041	<0.001^a^
Felt numb/detached from others	0–1	71	17.5	84	20.9	44.145	<0.001^a^

Notes

Two‐tailed paired‐samples *t* tests and chi‐square tests were used to compare participants’ scores on the main study variables at Time 1 and Time 2.

a Significant at the *p* < 0.05 level.

### 4.2 Correlates of new graduate health outcomes

As shown in Table 4, good mental health was positively associated with PsyCap (*r* = 0.28), person‐job fit (*r* = 0.27) and occupational coping self‐efficacy (*r* = 0.20). Weaker positive associations were found with new graduate support (*r* = 0.18), structural empowerment (*r* = 0.14) and authentic leadership (*r* = 0.12). Good mental health was negatively related to burnout (*r* = −0.32 and −0.33 for emotional exhaustion and cynicism, respectively) and work interference with personal life (*r* = −0.23). Incivility from all sources (*r* = −0.12 [physician], −0.14 [co‐worker] and 0.15 [supervisor]) was also significantly negatively related to mental health.

**Table 4:** Pearson's *r* correlations between Time 1 predictors and Time 2 health outcomes

	1	2	3	4	5	6	7	8	9	10	11	12	13	14
Time 1														
1. Authentic leadership	–													
2. Empowerment	0.50 (<0.001)	–												
3. Person‐job fit	0.50* (<0.001)	0.55* (<0.001)	–											
4. New graduate support	0.49* (<0.001)	0.52* (<0.001)	0.57* (<0.001)	–										
5. Occupational coping self‐efficacy	0.22* (<0.001)	0.28* (<0.001)	0.28* (<0.001)	0.29* (<0.001)	–									
6. Psychological capital	0.30* (<0.001)	0.41* (<0.001)	0.48* (<0.001)	0.52* (<0.001)	0.53* (<0.001)	–								
7. Work‐life interference	−0.17* (0.001)	−0.24* (<0.001)	−0.39* (<0.001)	−0.25* (<0.001)	−0.25* (<0.001)	−0.28* (<0.001)	–							
8. Supervisor incivility	−0.49* (<0.001)	−0.26* (<0.001)	−0.44* (<0.001)	−0.35* (<0.001)	−0.07 (0.185)	−0.12* (0.016)	0.25* (<0.001)	–						
9. Co‐worker incivility	−0.19* (<0.001)	−0.16* (0.002)	−0.43* (<0.001)	−0.38* (<0.001)	−0.06 (0.238)	−0.20* (<0.001)	0.25* (<0.001)	0.42* (<0.001)	–					
10. Physician incivility	−0.08 (0.130)	−0.09 (0.085)	−0.33* (<0.001)	−0.15* (0.002)	0.02 (0.709)	−0.15* (0.002)	0.22* (<0.001)	0.35* (<0.001)	0.46* (<0.001)	–				
11. Emotional exhaustion	−0.18* (<0.001)	−0.29* (<0.001)	−0.48* (<0.001)	−0.34* (<0.001)	−0.28* (<0.001)	−0.36* (<0.001)	0.57* (<0.001)	0.25* (<0.001)	0.33* (<0.001)	0.36* (<0.001)	–			
12. Cynicism	−0.21* (<0.001)	−0.35* (<0.001)	−0.52* (<0.001)	−0.40* (<0.001)	−0.28* (<0.001)	−0.43* (<0.001)	0.51* (<0.001)	0.27* (<0.001)	0.32* (<0.001)	0.33* (<0.001)	0.70* (<0.001)	–		
Time 2														
13. Mental health symptoms	0.12* (0.020)	0.14* (0.006)	0.27* (<0.001)	0.18* (<0.001)	0.20* (<0.001)	0.28* (<0.001)	−0.23* (<0.001)	−0.15* (0.003)	−0.14* (0.005)	−0.12* (0.020)	−0.32* (<0.001)	−0.33* (<0.001)	–	
14. Overall health	0.04 (0.472)	0.06 (0.217)	0.11* (0.030)	0.09 (0.072)	0.21* (<0.001)	0.17* (0.001)	−0.22* (<0.001)	−0.05 (0.321)	−0.13* (0.010)	−0.13* (0.013)	−0.20* (<0.001)	−0.25* (<0.001)	0.36* (<0.001)	–
15. PTSD risk (3/4 symptoms)	−0.16* (0.015)	−0.16* (0.030)	−0.27* (<0.001)	−0.18* (<0.001)	−0.20* (0.007)	−0.25* (<0.001)	0.29* (<0.001)	0.20* (0.004)	0.26* (<0.001)	0.180* (<0.001)	0.32* (<0.001)	0.34* (<0.001)	−0.47* (<0.001)	−0.22* (0.001)

*p*‐values for each correlation are provided in brackets.

*Significant, *p* < 0.05.

Overall health had fewer significant relationships with the predictor variables. Occupational coping self‐efficacy was most strongly related (*r* = 0.21). PsyCap (*r* = 0.17) and person‐job fit (*r* = 0.11) had weak significant correlations. Negative relationships were found between overall health and cynicism (*r* = −0.25), work interference with personal life (*r* = −0.22) and emotional exhaustion (*r* = −0.20) and co‐worker and physician incivility (*r* = −0.13 for both).

### 4.3 PTSD risk

Biserial correlations showed that Time 2 presence of symptoms of PTSD risk was significantly related to most major study variables except authentic leadership and new graduate nurse support. The presence of symptoms of PTSD risk had significant negative correlations with structural empowerment (*r* = −0.13), person‐job fit (*r* = −0.19), occupational coping self‐efficacy (*r* = −0.18) and PsyCap (*r* = −0.18). Positive relationships were found between the presence of symptoms of PTSD risk and work–life interference (*r* = 0.24), supervisor incivility (*r* = 0.17), co‐worker incivility (*r* = 0.20), physician incivility (*r* = 0.26), emotional exhaustion (*r* = 0.28) and cynicism (*r* = 0.28).

### 4.4 Regression analysis results

Table 5 gives multiple linear regression results for mental and overall health and logistic regression results for PTSD risk at Time 2. Occupational coping self‐efficacy (*β* = 0.15) and cynicism (*β* = −0.17) were significant predictors explaining unique variance in Time 2 mental health, with the model explaining 10.4% of the variance in mental health. For overall health at Time 2, cynicism was a significant predictor (*β* = −0.15), with the overall model explaining 15% of the variance in overall health. The effect size [Cohen's *f* ^2^ (Cohen, 1988)] was 0.11 for the mental health symptoms model and 0.18 for the overall health model. Power calculations were conducted using G*Power (*F* tests, Linear multiple regression: Fixed model, *R* ^2^ deviation from zero) with these effect sizes, *α* = 0.05, *N* = 406 and 12 predictors. Results showed that we achieved 99% power for both models.

**Table 5:** Standardized linear regression results

Time 1 independent variable	Mental health symptoms (Time 2)			Overall health (Time 2)		
	*β*	*SE*	*p*	*β*	*SE*	*p*
New graduate support	−0.037	0.090	0.597	−0.045	0.067	0.503
Authentic leadership	−0.002	0.051	0.981	−0.028	0.037	0.664
Structural empowerment	−0.030	0.017	0.650	−0.057	0.013	0.363
Person‐job fit	−0.079	0.113	0.315	0.112	0.081	0.124
Psychological capital	0.037	0.068	0.600	0.130*	0.051	0.050
Occupational coping self‐efficacy	0.145*	0.068	0.019	0.053	0.050	0.359
Supervisor incivility	0.021	0.042	0.750	−0.049	0.031	0.421
Co‐worker incivility	−0.089	0.040	0.158	−0.003	0.030	0.956
Physician incivility	−0.049	0.031	0.420	0.039	0.023	0.499
Emotional exhaustion	0.017	0.032	0.822	−0.117	0.024	0.107
Cynicism	−0.174*	0.032	0.021	−0.145*	0.024	0.044
Work–life interference	−0.117	0.028	0.061	−0.027	0.021	0.648
Final model *R* ^2^	0.104			0.150		

*Significant, *p* ≤ 0.05.

Logistic regression results presented in Table 6 shows that cynicism (*β* = 0.25) and work interference with personal life (*β* = 0.24) were significant predictors of PTSD risk, increasing the odds of PTSD risk by 28% and 27%, respectively. The overall model explained 17% of the variance in PTSD risk (Naglekerke's *r* square = 17.0). In logistic regression, odds ratios represent the effect size for each predictor (Tabachnick & Fidell, 2013). Power analysis in G*Power (z tests: Logistic regression: Post hoc: Compute achieved power**—**given alpha, sample size and effect size) determined that we had 79% power to detect an odds ratio of 1.28 (cynicism) and 77% power to detect an odds ratio of 1.27.

**Table 6:** Logistic regression results

	*B*	*SE*	Wald	Sig.	Exp(*B*)
New graduate support	−0.012	0.356	0.001	0.973	0.988
Authentic leadership	−0.079	0.204	0.152	0.697	0.924
Structural empowerment	0.081	0.071	1.317	0.251	1.085
Person‐job fit	−0.348	0.443	0.617	0.432	0.706
Psychological capital	−0.347	0.281	1.524	0.217	0.707
Occupational coping self‐efficacy	−0.001	0.279	0.000	0.998	0.999
Supervisor incivility	−0.046	0.155	0.088	0.766	0.955
Co‐worker incivility	0.262	0.151	3.014	0.083	1.300
Physician incivility	−0.105	0.123	0.732	0.392	0.900
Emotional exhaustion	0.002	0.137	0.000	0.991	1.002
Cynicism	0.250*	0.124	4.071	0.044	1.284
Work–life interference	0.238*	0.115	4.278	0.039	1.268
Naglekerke's *r* ^2^	17.0				

Naglekerke's *r* ^2^ gives a measure of the explanatory power of the logistic regression model (Nagelkerke, 1991).

*Significant, *p* < 0.05.

## 5 DISCUSSION

This research contributes to the literature about new graduate nurses’ health outcomes during their transition to practice in several key ways. First, the results represent a national sample of nurses from across Canada and demonstrated that both situational and personal factors were significantly related to new graduate nurses’ health outcomes, supporting the New Graduate Successful Transition Retention Model. The findings of this study also showed that, overall, early career nurses perceived themselves to be in good mental and overall health. It was troublesome to see that over 20% were at risk of PTSD as a result of experienced workplace mistreatment, despite overall reports of low frequencies of incivility at work. However, these results are valuable for nurses, managers and organizations who are interested in understanding the concerns and issues facing new graduate nurses early on in their careers.

As expected, correlational analysis revealed that positive work environment factors were associated with better health outcomes for new graduate nurses. Person–job fit with six areas of work–life stood out as being particularly important because it was significantly related to all three health outcomes. This is consistent with a previous study by Laschinger and Grau (2012) which linked new graduate nurses’ perceptions of person‐job fit to both their mental and physical health. In their study, emotional exhaustion was found to mediate the influence of person‐job fit on physical health symptoms, while bullying and cynicism mediated its impact on mental health symptoms. Among a general sample of staff nurses, Laschinger and Finegan (2005) also linked person‐job fit to physical and mental health symptoms, a relationship that was again mediated by burnout. Our findings suggest that the match between new graduate nurses’ work environment and their expectations has an important influence on their health during their first years of practice. Thus, new nurses and organizations should consider person‐job fit when making career and staffing decisions.

Authentic leadership and structural empowerment were related to mental health and PTSD risk but, surprisingly, not to overall health. Few studies have specifically examined the influence of these situational factors on new graduate nurses’ health outcomes. Evidence suggests that factors external to the work environment, such as, exercise, nutrition and sleep, are important predictors of nurses’ health (Sveinsdóttir & Gunnarsdóttir, 2008), which points to the need to examine these factors in future studies. Moreover, participants also rated their overall health highly, suggesting that few were experiencing significant health problems early on in their career.

Previous research has linked authentic leadership and structurally empowering working conditions to positive outcomes for nurses during the early stages of their careers. For example, Read and Laschinger (2013) found that both authentic leadership and structural empowerment were significantly related to mental and physical health symptoms among new graduate nurses in Ontario. In a separate study, Laschinger and Fida (2014b) showed that authentic leadership had a positive effect on new graduate nurses’ mental health by reducing burnout. Other studies have linked structural empowerment to physical and mental health of staff nurses (Laschinger & Finegan, 2005) and front‐line and middle nurse managers (Laschinger, Almost, Purdy, & Kim, 2004).

Personal factors were also significantly related to new graduate nurses’ health in this study. PsyCap and occupational coping self‐efficacy were significantly related to all three health outcomes, highlighting the important role that new graduate nurses’ intrapersonal resources play in protecting their health, thought to be related to their ability to handle work‐related stressors (Laschinger & Grau, 2012; Laschinger et al., 2013, 2015). Findings add to growing evidence that development of new graduate nurses’ intrapersonal resources contributes to their health and well‐being during their stressful transition to practice. The results also suggest that to support a healthy nursing workforce, nurse leaders should pay close attention to and work to develop new graduate nurses’ self‐confidence to perform and learn, hope about the future, an optimistic outlook on work‐related events and situations and resiliency to overcome challenges and accomplish goals. Furthermore, as suggested by Duchscher (2008) and others (Casey et al., 2004; Scott et al., 2008) new graduate nurses need transition support and help developing realistic expectations of themselves during their first years as a professional nurse. The result that new graduate support was related to better mental health and fewer PTSD symptoms adds empirical support to this contention.

Incivility from co‐workers and physicians was significantly negatively related to all three health outcomes, while supervisor incivility was important for mental health and PTSD risk, but not overall health. While it was encouraging to see that nurses reported low incidences of incivility, this finding suggests that incivility does not have to occur frequently to have detrimental effects on new graduate nurses’ health. The link between workplace mistreatment and nurses’ health has been supported by past research (McKenna, Poole, Smith, Coverdale, & Gale, 2003; Quine, 2001; Read & Laschinger, 2013). In a study of Ontario new graduate nurses, Wing et al. (2015) showed that both co‐worker and supervisor incivility, while infrequent, were significantly related to increased mental health symptoms. In another study, Laschinger et al. (2013) also found that overall incivility was infrequent but was a significant factor influencing new graduate nurses’ mental health. Thus, workplace incivility remains a concerning problem affecting newcomers to the nursing profession.

Post‐traumatic stress disorder risk resulting from workplace violence is not uncommon in nursing and has been linked to negative health outcomes for nurses and healthcare organizations. For instance, in a study of emergency department nurses Gates, Gillespie, and Succop (2011) found that 91% had PTSD symptoms after experiencing workplace violence from patients and visitors and that these symptoms were associated with increased stress and decreased work productivity. Among new graduate nurses, McKenna et al. (2003) found that experiencing workplace violence from colleagues and supervisors led to feelings of sadness, depression, anxiety, fear and nervousness. In a previous study, Laschinger and Nosko (2015) also found that workplace bullying was significantly related to PTSD symptoms among both novice and experienced nurses. Findings linking incivility to new graduate nurses’ PTSD risk add to this research by demonstrating that incivility, while thought to be less severe than bullying, also appears to be psychologically harmful to new graduate nurses.

The results also highlighted the positive association between burnout and new graduate nurses’ health, with significant relationships between both components of burnout and each of the health‐related outcomes. While the link between burnout and new graduate nurses’ mental (Laschinger & Grau, 2012; Laschinger et al., 2015) and physical health (Laschinger & Grau, 2012) has been reported in previous studies, this is the first to show that burnout is positively related to increased risk of PTSD in this population. This result demonstrates the importance of reducing and preventing burnout among new nurses to prevent its damaging effects on mental health.

### 5.1 Predictors of new graduate nurses’ health outcomes

Few variables remained significant predictors in the regression analyses. Cynicism was a significant predictor of all three health outcomes, while occupational coping self‐efficacy explained unique variance in mental health and work–life interference explained unique variance in PTSD risk.

The finding that occupational coping self‐efficacy was particularly important for mental health suggests that new graduate nurses’ who believe that they can cope with the demands of their new role are better able to handle work stress, which, in turn, positively influences their mental health. This is consistent with past research (Laschinger et al., 2015) and suggests that development of new graduate nurses’ capacity to cope with job demands is particularly important for nurse managers. Furthermore, work–life imbalance may cause significant stress, leading to symptoms of depression and PTSD such as nightmares, constant thoughts about work and feeling constantly on edge. Given the detrimental effects of work–life imbalance on new nurses’ health and job performance, and their perceptions of nursing as a career, it is important for managers to create reasonable workloads and ensure that resources are in place that empower new nurses to be effective and satisfied at work.

Cynicism was a significant predictor of all three health outcomes, strengthening the growing body of evidence showing the negative effects of burnout on the health and well‐being of new graduate nurses (Laschinger et al., 2010; Rudman et al., 2014). Burnout prevention strategies addressing both personal coping capabilities and contributing work environment factors may help reduce cynicism. Awa, Plaumann, and Walter (2010) found that combining person‐directed interventions, such as cognitive behavioural therapy to enhance personal coping skills, and organization‐directed interventions, such as improving workloads and giving employees more autonomy or more involvement in decision‐making, were most effective for preventing burnout. Therefore, providing a range of tailored person‐ and organization‐directed interventions is needed to reduce the likelihood of burnout among new graduate nurses. In addition, the positive outcomes associated with burnout interventions wane after 6 months to a year; therefore, ongoing assessment and periodic refresher sessions are needed for long‐term burnout management (Maslach, Leiter, & Jackson, 2012).

### 5.2 Avenues for future research

The results of the current study suggest that the development and testing of workplace interventions to promote the mental and physical health of new graduate nurses is an important area to investigate further. Interventions that target authentic leadership development and focus on managers’ role along with practical strategies to create structurally empowering work environments, promote civility at work and cultivate PsyCap among new graduate nurses may be particularly effective in promoting positive health outcomes in this employee group. There may also be important subgroups in our sample who are at higher or lower risk of developing mental and physical health problems. Future research with a larger or stratified sample could investigate this further.

### 5.3 Limitations

Limitations to this study include the low survey response and the use of self‐report questionnaires. Self‐report measures are susceptible to response bias due to an individual's tendency to respond in socially appropriate ways (Donaldson & Grant‐Vallone, 2002). However, strategies were implemented in an attempt to mitigate social desirability. For example, the current study did not ask sensitive questions and participants were also informed that they could skip questions that they did not wish to answer. Lastly, participants were able to complete these confidential surveys in a private setting (such as their own home), which reduces the fear of retribution from their place of employment.

The low response rate at Time 1 may have reduced generalizability of results due to non‐response bias. As shown in the results, we had high power for our linear regression analyses but less than desirable power in our logistic regression model; therefore, this analysis would have benefited from a larger sample. Evidence exists that survey response rates are falling among nurses (Auerbach, Staiger, Muench, & Buerhaus, 2012) due to greater time constraints and workloads (VanGeest & Johnson, 2011). It is possible that new nurses experiencing extreme stress and burnout may have been more reluctant to participate in the study. Thus, cautious interpretation of the generalizability of our findings is warranted.

The relationships between Time 1 predictors and Time 2 health outcomes had small to moderate effect sizes (Cohen, 1988); therefore, cautious interpretation of the results is warranted. This is not entirely surprising given that there are many factors outside of work that contribute to mental and physical health (Centers for Disease Control and Prevention, 2014).

Finally, although having a time‐lagged design gives stronger evidence of the causal relationship between variables in the model than a cross‐sectional design, the lack of a longitudinal research design could be considered another limitation of the study.

## 6 CONCLUSION

The results of the current study provide a look into the work–life experiences of newly graduate nurses in Canada over a 1‐year time period and identify factors that influence their health outcomes during their early career years. These findings show that although, overall, new graduate nurses feel positive about their work experiences and are healthy, there is still work to be done to improve working conditions for new graduate nurses, especially in regard to addressing workplace incivility and burnout. Promoting and sustaining the health and well‐being of new graduate nurses is essential for the health and sustainability of the nursing workforce and healthcare organizations more generally. These results highlight the important influence that the work environment and internal personal resources can have on new graduate nurses’ health outcomes. Thus, healthcare leaders play an invaluable role in developing and implementing strategies to create healthy, civil and satisfying work environments for new nurses and helping them develop the personal skills and strategies they need to cope with the demands of their new role.

## CONFLICT OF INTEREST

No conflict of interest has been declared by the authors.
